# On Laminated Object Manufactured FDM-Printed ABS/TPU Multimaterial Specimens: An Insight into Mechanical and Morphological Characteristics

**DOI:** 10.3390/polym14194066

**Published:** 2022-09-28

**Authors:** S. Kumar, I. Singh, S. S. R. Koloor, D. Kumar, M. Y. Yahya

**Affiliations:** 1 Department of Mechanical Engineering, CT University, Ferozepur Rd, Sidhwan Khurd, Ludhiana 142024, Punjab, India; 2 Institute for Structural Engineering, Department of Civil Engineering and Environmental Sciences, Universität der Bundeswehr München, Werner-Heisenberg-Weg 39, 85579 Neubiberg, Germany; 3 Centre for Advanced Composite Materials, Faculty of Engineering, School of Mechanical Engineering, Universiti Teknologi Malaysia, Johor Bahru 81310, Johor, Malaysia

**Keywords:** renewable polymers, laminated object manufacturing, FDM printing, polymer composite, acrylonitrile butadiene styrene, thermoplastic polyurethane, flexural strength, surface roughness, scanning electron microscopy

## Abstract

Fused deposition modeling (FDM) printing of commercial and reinforced filaments is a proven and well-explored method for the enhancement of mechanical properties. However, little has hitherto been reported on the multi-material components, fused or laminated together into a single specimen by using the laminated object manufacturing (LOM) technique for sustainable/renewable polymers. TPU is one such durable and flexible, sustainable material exhibiting renewable and biocompatible properties that have been explored very less often in combination with the ABS polymer matrix in a single specimen, such as the LOM specimen. The current research work presents the LOM manufacturing of 3D-printed flexural specimens of two different, widely used polymers available viz. ABS and TPU and tested as per ASTM D790 standards. The specimens were made and laminated in three layers. They were grouped into two categories, namely ABS: TPU: ABS (ATA) and TPU: ABS: TPU (TAT), which are functionally graded, sandwiched structures of polymeric material. The investigation of the flexural properties, microscopic imaging, and porosity characteristics of the specimens was made for the above categories. The results of the study suggest that ATA-based samples held larger flexural strength than TAT laminated manufactured samples. A significant improvement in the peak elongation and break elongation of the samples was achieved and has shown a 187% increase in the break elongation. Similarly, for the TAT-based specimen, flexural strength was improved significantly from approximately 6.8 MPa to 13 MPa, which represents a nearly 92% increase in the flexural strength. The morphological testing using Tool Maker’s microscopic analysis and porosity analysis has supported the observed trends of mechanical behavior of ATA and TAT samples.

## 1. Introduction

In recent years, additive manufacturing (AM) has been exponentially employed for different engineering applications, among other things. For polymeric processing of material matrixes, many AM techniques are common, such as stereolithography (SLA), digital light processing (DLP), fused filament fabrication (FFF), etc. [1,2,3]. Among these AM technologies, FFF has been extensively used for polymer processing due to its inbuilt capabilities of processing polymer with varying input parameters viz. infill percentage, infill angle, the number of perimeters, infill density, cooling rate, bed temperature, etc. [4,5]. Several polymeric materials (such as acrylonitrile butadiene styrene (ABS), polylactic acid (PLA), nylon (PA6), polypropylene (PP), polyether ether ketone (PEEK), low-density polyethylene (LDPE), high-density polyethylene (HDPE), etc.) have been used for numerous industrial and prototyping applications [6,7,8,9,10] on the FDM printing platform and are easily available in standard sizes on the commercial market. The commercially available materials have a major constraint in that they cannot exhibit all the desirable properties in one single material. Therefore, various research groups have used composites of polymers in which they have reinforced foreign particles of metal, non-metal, ceramics, polymeric fiber, natural fibers, nanopowders, etc. to obtain extensive mechanical properties [11,12,13,14,15].

Thermoplastic polyurethane (TPU) is one such polymeric matrix which has excellent mechanical properties, such as large elongation, moderate tensile and compressive strength, and better abrasion resistance. It is also renewable, sustainable, and biocompatible. The TPU, along with its excellent mechanical properties, is biocompatible and renewable, which makes it vital for AM in various applications [16]. One of the studies on TPU polymer has observed that the design consideration of sections under mechanical loading may play a vital role in the selection of the specimen. The study highlighted that the properties of TPU were not homogenous for different design considerations of sections as per different ISO standards [17]. TPU, being a polymeric material, has low thermal and electrical conductivity, but its properties may be altered by adding foreign reinforcement to the material matrix. One such study reported the use of a graphene nanolayer reinforcement in TPU, which resulted in increased electrical conductivity of the TPU matrix. The results of the study have shown that the solution blending method is the best technique by which to prepare the TPU/graphene composite and has shown linear electrical conductivity of 233 × 10^−8^ S/cm^2^ [18].

Various studies have used epoxy-based resins to prepare laminated object composites of polymers. One such study reports the use of epoxy-based resins for laminated object manufacturing of polymeric material to explore the adhesive strength of epoxy resins in the case of polymers [19]. Brancewicz et al. [20] reported on the adhesive strength of the epoxy resins for the PLA and TPU composites [20]. The reported studies on polymeric composites using epoxy resins have evaluated and observed the adhesive strength of the material by testing the single lap joint by using the typical ASTM D1002 testing standard [21]. It has been ascertained that the epoxy resins have adhesive strength in between the range of 10–15 MPa for a range of materials [19,20,21]. 

One of the studies reported the use of PLA microfibers in reinforced TPU materials at a ratio of 80:20 by using a twin-screw extrusion machine (TSE), and the blend was used to prepare in-house feedstock filament and was ultimately tested for mechanical, morphological properties. The results were compared with the compression molding process for the same TPU/PLA specimens, and it was observed that FDM-printed parts have shown better crystallinity above 24.92%, whereas for compression molding it was 18% [22]. 

A similar study on polymeric matrix TPU/PLA reinforced with graphene oxide to alter the properties of the polymeric matrix has been performed, and improvements in the mechanical and thermal stability were observed as output. The maximum compression modulus was reported for the 5 wt% loaded TPU/PLA (7:3) sample. The addition of graphene oxide in the TPU/PLA sample has led to a 167% increase in the compression modulus and a 76% increase in the tensile modulus. The TPU/PLA reinforced with 5 wt% graphene oxide has shown 57 MPa of tensile modulus with improved strain at break and a yield strength of the composite matrix [10]. TPU and polyamide blends have shown the renewable composites obtained by synthesizing dimers of fatty acids and the reported composite has shown a positive change in the material characteristics of parent polymers. The reinforcement of polyamide in the TPU had shown improvement in the mechanical properties such as Young’s modulus and yield stress of the material with increased loading of polyamide [23]. The biodegradability and renewability of the TPU polymer make this material special for biomedical and nanodrug-loading applications.

Wang et al. [24] reported the possibility of the shape memory effect of the PCL matrix reinforced with the TPU matrix. The study observed that the TPU reinforcement affected the mechanical properties significantly. There was a large-scale reduction in the tensile strength of the PCL when reinforced with TPU. The 3D-printing parameters in the study had hardly any effect on the shape memory behavior of the polymeric matrix [24]. TPU has shown large flexibility for FDM printing and biocompatibility for different biomedical applications. The TPU filament-based specimen has shown biocompatibility for NIH 3T3 cells, whereas medical-grade TPU (S-TPU) has shown greater compatibility for 3T3 cells than filament prepared by using medical-grade TPU (F-TPU) [25]. Ritzen et al. [26] have explored and established the self-healing properties of TPU. The study suggested that the TPU material has shown durability and long retention of self-healing characteristics even after passing through different heating cycles. 

Abeykoon et al. [27] tested five different commercially available feedstock filaments of PLA, ABS, carbon fiber PLA (CFPLA), CF-ABS, and carbon nanotube ABS with varying 3D-printing input process parameters. The study quantified that the bending modulus for the PLA and ABS was approximately 974 and 550 MPa, whereas the highest bending modulus of 1250 MPa was observed for CF-PLA. From the SEM images, it has been reported that the 90 mm/s speed of 3D printing has resulted in the best arrangement of a layer inside the specimen [27]. Markiz et al. [28] studied the impact of infill printing direction for ABS specimens. The study observed that the infill angle of 0° has resulted in a maximum tensile strength of 22 MPa, whereas 90 degrees of orientation has shown poor results of 12 MPa of tensile strength of the ABS specimens. 

One such study reports the anisotropic behavior [29] of ABS and polycaprolactone (PC) material matrix for tensile and shear properties. The results of the study noted that the largest anisotropy was prevalent for the strain energy densities of 3D-printed material with ±45° flat orientation in comparison to ±45° upright build orientation [30]. Baich and Manogharan [31] studied the effect of infill print parameters on the mechanical properties of 3D printing of ABS specimens and related it to the cost-effectiveness of the selected process parameter. Perez et al. [32] reinforced TiO_2_ in the ABS matrix, and the results suggested that flat orientation with 5 wt% reinforced TiO_2_ resulted in maximum tensile strength of 32 MPa. The reinforcement of elastomeric material in the ABS matrix has shown better morphological characteristics than pure ABS. In the same study, ABS was also reinforced with jute fiber, which resulted in a 9% decrement in tensile properties of ABS material [32]. 

Li et al. [33] have reviewed several techniques of 3D printing for the functional gradation of specimens. The study has suggested various ways of increasing the functionalities of 3D-printed specimens by (a) the print pause print (PPP) approach for multi-material printing, (b) inserting pre-printed parts in a specific location in other parts, and (c) multi-material printing using a suitable technique. Kumar et al. [34] established a relationship between the mechanical properties of ABS, PLA, and a single specimen comprising ABS, high-impact polystyrene (HIPS), and PLA alternative layers. The study reported that when ABS, PLA, and HIPS were combined in one specimen at the alternate position, the layer position played a significant role, and the properties observed were intermediate to the parent polymer properties. Similar studies have been reported by Yadav et al. [35] in which ABS and polyethylene terephthalate glycol (PETG) use 50%:50% in an alternate layer fashion. The study has highlighted that 50% of infill density has shown maximum tensile strength of 33 MPa which is very near to the tensile strength of the parent polymeric material. A further increase in infill density has shown a reduction in tensile strength of the multi-material component. Table 1 shows the previous studies performed based on LOM study and differentiates the present study from the performed studies.

## 2. Problem Statement and Objective of the Research

Past studies have revealed that ABS and TPU materials are widely explored for FDM printing applications due to their renewable properties/sustainability, durability, mechanical strength, and many other characteristics. The literature also highlighted that the TPU was extensively explored for engineering, non-structural engineering, and biomedical applications due to its renewability, approximability, biocompatibility, and biodegradability. However, little has hitherto been reported on the multilateral component of ABS and TPU in a single specimen by using laminated object manufacturing (LOM). ABS is a stiffer material and TPU, being a flexible, biocompatible, renewable [40,41,42,43,44] material when combined in a single specimen by using the LOM technique, may be used in applications where flexibility and stiffness are required in an adequate ratio. The scarcity of relevant data for multi-material components of polymeric structures, especially ABS and TPU widely used for 3D printing motivated the authors to deal with such a problem. Therefore, the present study aims to investigate a multi-material ABS/TPU test specimen prepared of different polymers by using FDM technology and to study the mechanical and morphological behavior in contrast to the individual ABS and TPU material. 

## 3. Materials and Methods

### 3.1. Materials

In the present study, an effort has been made to prepare a LOM test specimen by using the combination of renewable and sustainable TPU material with the stiff and strong ABS material matrix as per ASTM D790 standard. For FDM printing, the material ABS and TPU feedstocks of Ø1.75 mm were procured from Wanhao (Zhejiang, China) and Solidspace Technology LLP (Nashik, India), and an Amazon Dream polymer (Gujrat, India) feedstock supplier, respectively.

### 3.2. 3D Printing

**Figure 1:**
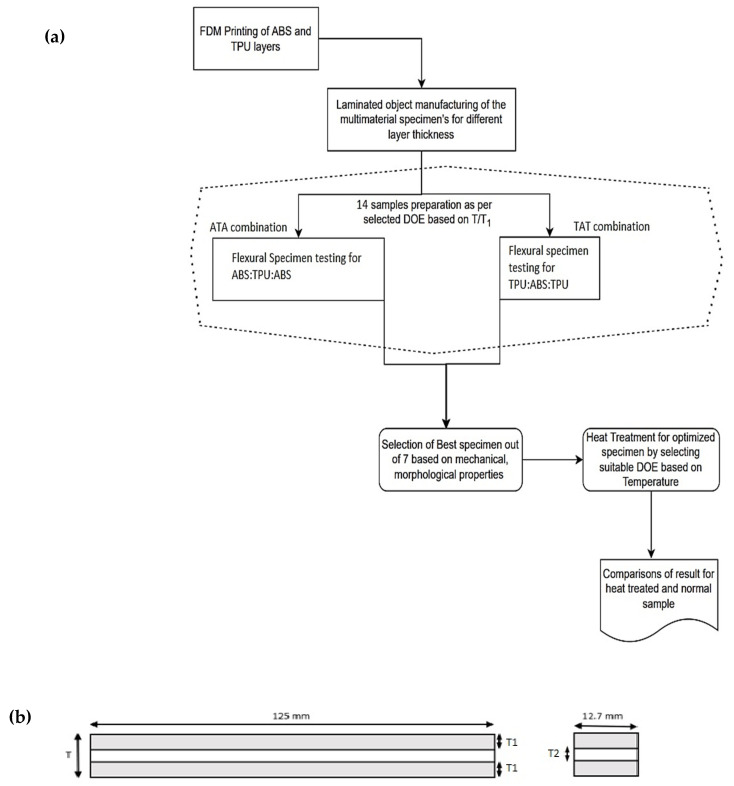
(**a**) Laminated object manufacturing of ABS/TPU specimens for exploration of mechanical and morphological properties. (**b**) Schematic view of multi-material bending specimen as per ASTM D790.

For FDM printing of ABS and TPU, an open-source FDM printer (divide by zero) (Make: Shenzhen Creality 3D Technology Co., Ltd., Shenzhen, China) was used with 3D slicer software (Ultimaker Cura 4.1; Utrecht, Netherlands). All the test specimens were printed with similar input parameters listed in Table 2. Figure 1 shows the (a) methodology used in the current study and (b) schematics of the specimen as per ASTM D790 standards.

### 3.3. Selection for the Sample Design or Sandwiched Structure 

**Figure 2:**
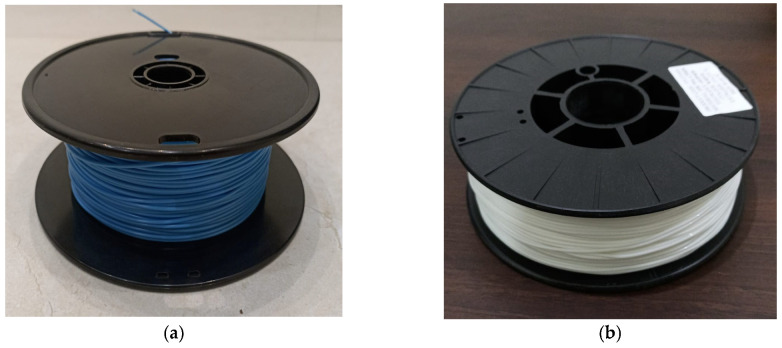
Feedstock filaments for FDM printing (**a**) ABS and (**b**) TPU.

To study the influence of object lamination in different layers, a dimensionless parameter T/T1 (where T is the thickness of full flexural specimen and T1 is the thickness of material outside the sandwiched structure) has been taken as one of the important criteria for 3D printing. The T/T1 ratio varied from 2.25 to 3.75 for both the combinations to evaluate the impact of the thickness of outer material (T1) in the multi-material specimen, which was used as outer layers for other material having thickness T2. Figure 2 shows the feedstock filaments for (a) ABS and (b) TPU material. 

**Figure 3:**
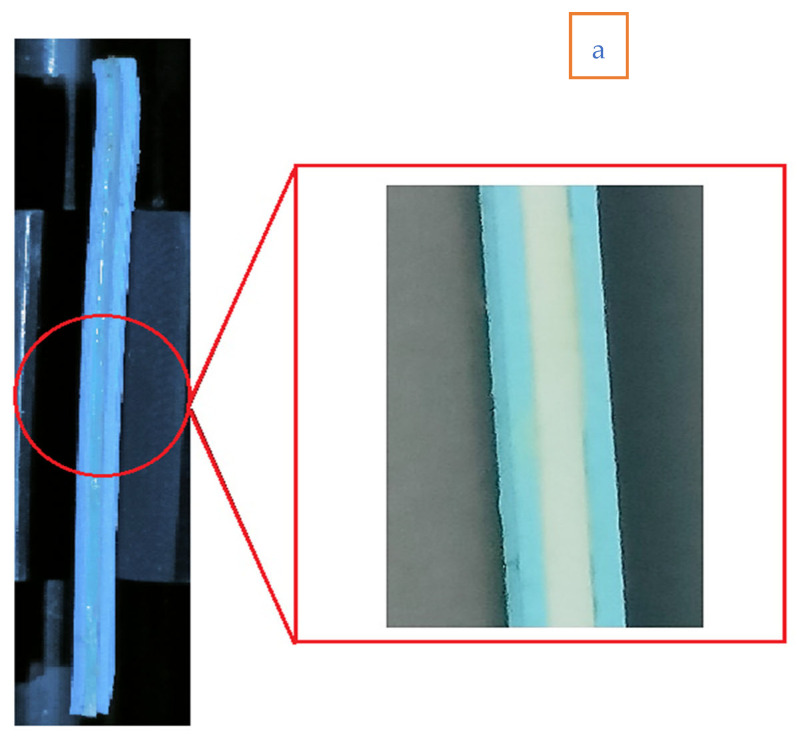

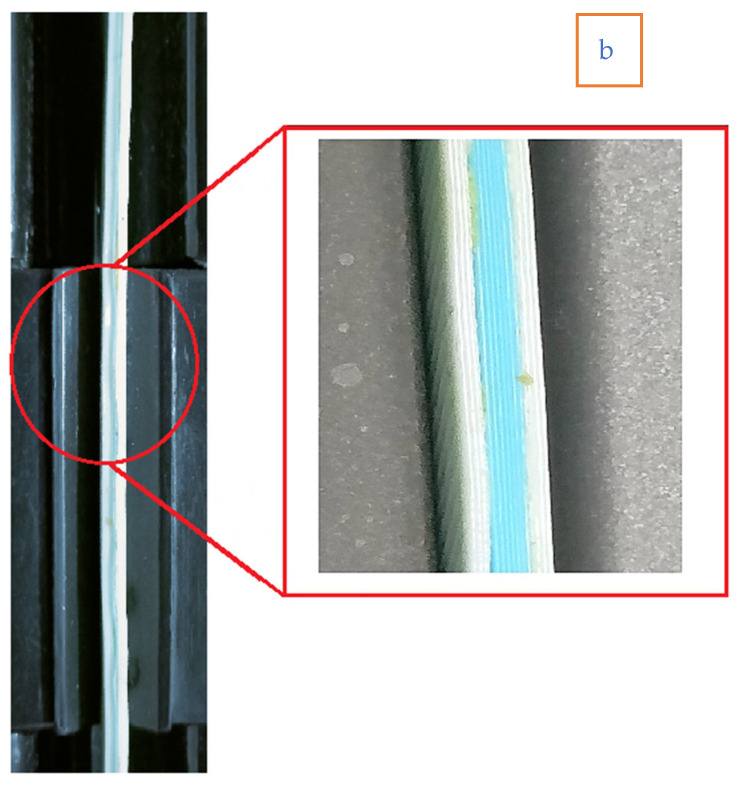
Flexural sample for ABS and TPU material (**a**) ATA and (**b**) TAT combination.

Figure 3a shows ATA (ABS: TPU: ABS) combination and Figure 3b shows the TAT (TPU: ABS: TPU) combination of the specimen. Table 3 shows the specimen thickness ratio (T/T1) and T2 for ABS and TPU material.

### 3.4. Standard Testing and Details

The adhesive bond strength between the two polymers was already established by using the ASTM D1002 standard by preparing three samples for the lap joint. Shear properties of the adhesive joint were tested by a universal testing machine. The samples were loaded in tension at a deformation speed of 2 mm/min (according to ASTM D1002 standard) at room temperature. The specimen’s adhesive strength was found to be satisfactory (adhesive strength 12 ± 1 MPa). Therefore, all of the specimens were prepared by using the same technique and epoxy resin.

**Figure 4:**
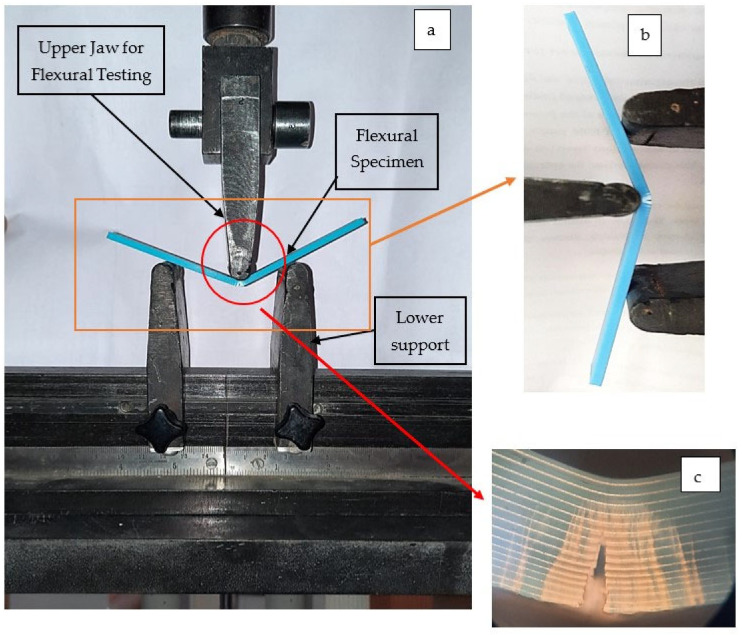
(**a**) UTM setup for testing for flexural specimen using ASTM D790 standard, 4 (**b**) focused area under flexural testing, and 4 (**c**) magnified image for the fractured area of ABS specimen.

To prepare two different kinds of LOM sandwiched structures (a) ATA (ABS: TPU: ABS) and (b) TAT (TPU: ABS: TPU), 14 different samples were prepared along with two samples of full ABS and full TPU for comparison. After the preparation of the flexural specimen as per ASTM D790 standard, the UTM machine (Make: Shanta Engineering, Mohali, India) has been used for flexural testing (see Figure 4) by using load cell 1KN and an extensometer (Make: AVE639; Dakseries; Make: Instron, Chennai, India). The flexural testing was performed by using a 20 mm/s testing speed. The tested samples were then cut from the middle section and then cut samples were used for morphological analysis such as Tool Maker’s microscopic images for porosity analysis, SEM analysis, and 3D rendering of SEM images. 

## 4. Results and Discussion

### 4.1. Flexural Testing Results for ATA Specimens

Table 4 and the results obtained from the study for pure ABS and TPU specimens. Table 5 and Table 6 depict the flexural properties of ATA (ABS: TPU: ABS) and TAT (TPU: ABS: TPU) functionalized polymeric specimens, respectively. From the results, it may be observed that among the ABS and ATA-based specimens’ the maximum flexural strength of 64.16 ± 0.41% MPa was observed for ABS. The results also suggest that with the addition of TPU layers inside the ABS there has been a significant decrease in the flexural strength. 

For the maximum thickness of 1.54 mm of TPU in the ATA specimen (S.No. 7, Table 5), the least flexural strength of 31.38 ± 0.21% MPa has been observed. This may be due to the presence of more volume of TPU between ABS layers which exhibit weak flexural properties. However, sample 4, with an equal thickness of three layers of 1.10 mm (S.No. 4, Table 5) has shown maximum flexural strength of 46.49 ± 0.35% MPa for ATA specimens. Thus, for better flexibility and moderate flexural strength, a layer thickness of 1.10 mm may be used. The single layer of TPU in the ABS specimens has increased the break and peak elongation significantly. For sample 7 (S.No. 7, Table 5), there has been a 327% significant increment in the break elongation of ATA specimen in comparison to pure ABS specimen. 

**Figure 5:**
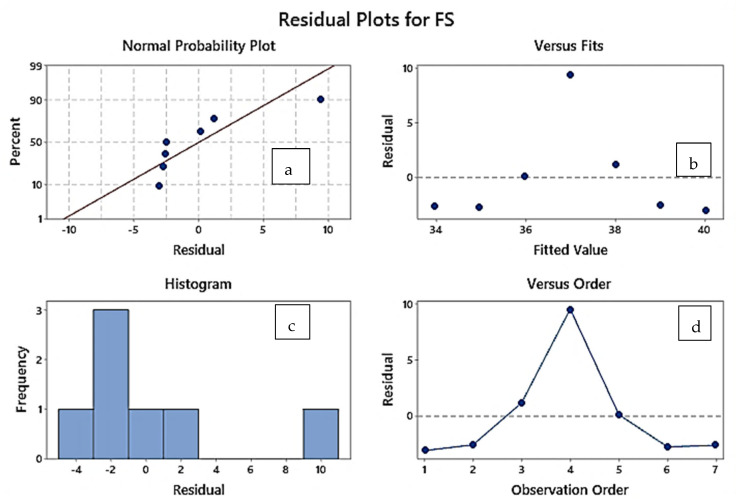
Residual plot for ATA samples based on a linear regression model. (**a**) Normal probability plot, (**b**) Fit vs. residual plot. (**c**) Frequency vs. residual plot. (**d**) Residual vs. observation order plot.

For sample 4 (S.No. 4, Table 5), which held the best flexural strength among the ATA material-based specimens, the percentage increase in the break elongation was observed to be 187%. Figure 5 shows the residual plots vs. fit based on the regression model for data in Table 5 specially plotted for the flexural strength values of ATA samples (S.No. 1 to S.No. 7). From Figure 5a, it may be observed that the data lies near the normal line and the fourth reading outperforms, which was also the best in the case of flexural strength. 

**Figure 6:**
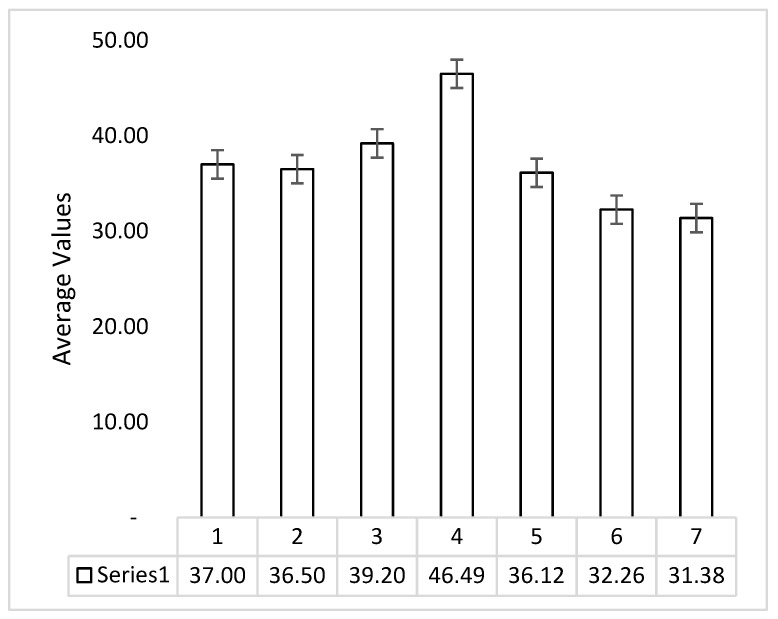
Error bar graph for flexural strength of ATA specimen for three samples of each group.

Three samples for each setting were prepared and tested so that average values may be reported for the study. Figure 6 shows the error bar for the process data of ATA specimens obtained from UTM testing for flexural strength. In Table 5, all average values of the samples have been reported. The average standard deviation for flexural strength was observed to be 1.82. Similarly, the standard deviation for the strain and Young’s modulus was observed to be valued were observed to be 0.08 and 0.23, respectively.

### 4.2. Flexural Testing Results for TAT Samples

Table 6 shows the flexural properties of TPU and TAT (TPU: ABS: TPU) specimens. From the results, it may be observed that TPU held flexural strength of 6.8 MPa, which is near to the data reported by previous researchers [45]. From the results, it may be observed that the addition of ABS layers inside the TPU structure leads to an increase in the flexural strength of the samples. From Table 6, it may be observed that maximum flexural strength of 12.92 ± 0.56% MPa was observed for sample 14 (S.No. 14, Table 6). This may be due to the addition of the highest-thickness ABS layer in sample 14, which gave it strength to resist the applied load. There was a significant decrease in the break elongation of the samples with increasing ABS thickness in the TAT specimen. However, it was evident from the test results that there was no steep decrease in the break elongation and peak elongation values in different TAT specimens.

**Figure 7:**
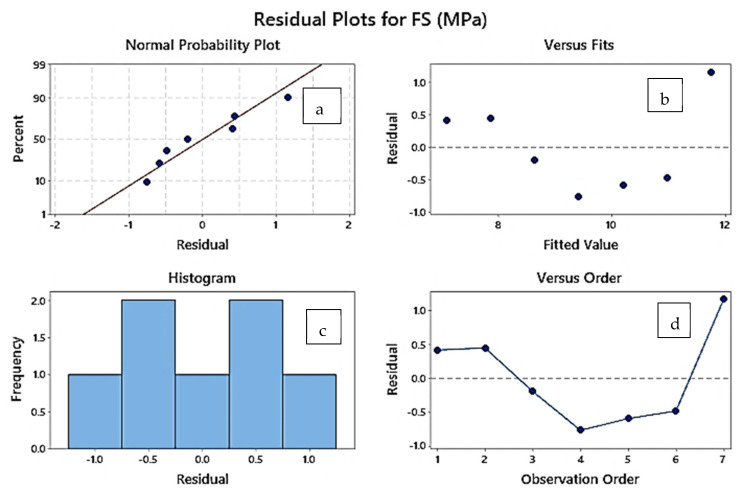
Residual plot for TAT samples based on a linear regression model. (**a**) Normal probability plot. (**b**) Fit vs. residual plot. (**c**) Frequency vs. residual plot. (**d**) Residual vs. observation order plot.

The TAT samples have observed a single trend of increasing flexural and break strength from sample 8 to sample 14 (see Table 6), whereas for the peak elongation and break elongation reversal trend was observed. Figure 7 shows the residual plot for the TAT samples (S.No. 8–14, Table 6), which depicts that with an increase in the layer thickness of ABS inside the TPU structure the flexural strength got improved significantly. Figure 7a shows one residual plot for TAT samples, indicating that the data was normal as the values lay close to the normal plot.

**Figure 8:**
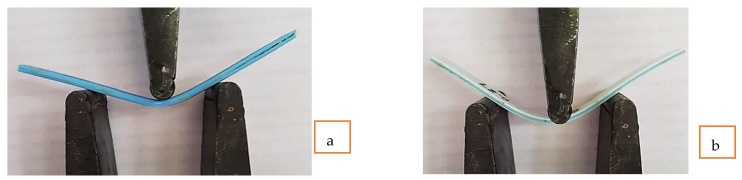
Flexural testing of specimens (**a**) V-shaped bending for ATA specimen (S.No. 4). (**b**) U-shaped bending of the specimen made of TAT material.

The tested sample for flexural specimens had shown flexibility and significant strain energy absorbance when ABS and TPU material layers were sandwiched between each other. Maximum strain absorbance has been observed for the samples made of TAT specimens (S.No. 14, sample 14), which is evident from the real-time flexural testing as shown in Figure 8. The TAT samples have shown U-shaped bending and, in some cases, no fracture has been observed. However, in the case of ATA samples (S.No. 4, Sample no. 4), V-shaped bending was prevalent due to greater stiffness than the TAT specimens. 

**Figure 9:**
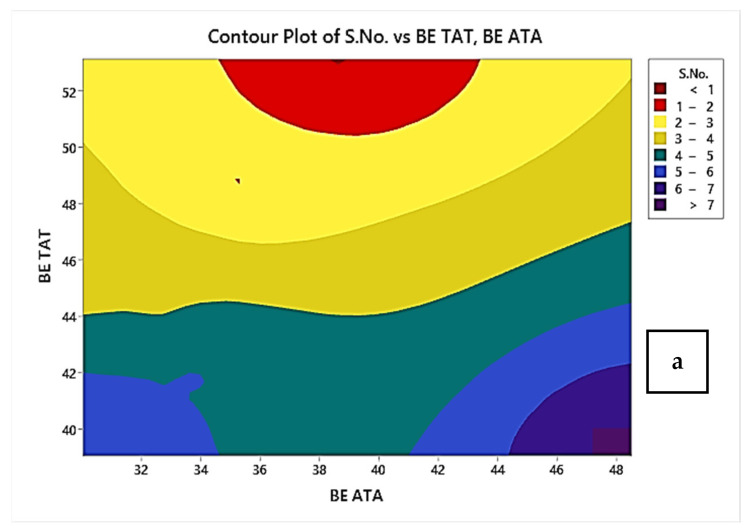

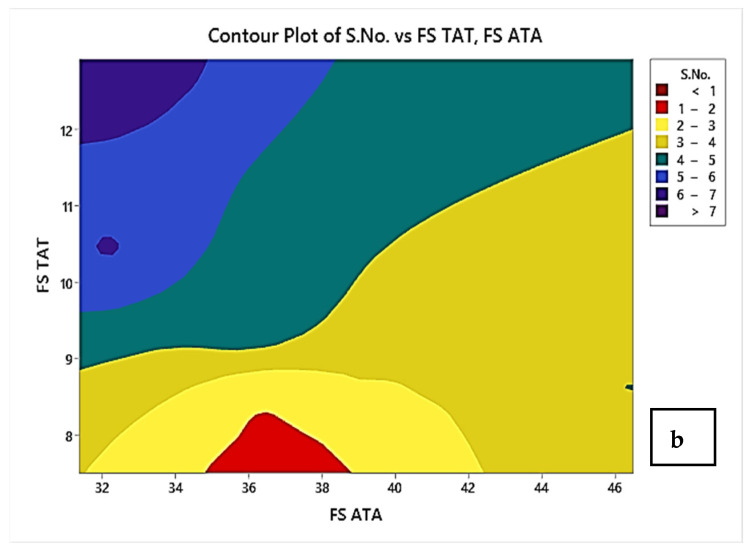
Two-dimensional contour plots for (**a**) break elongation and (**b**) flexural strength of ATA and TAT samples.

Figure 9a shows the contour plots for break elongation of TAT- and ATA-based samples. From the contour plot, it may be observed that for the ATA-based sandwiched structure as the T_2_ thickness increased in a dimensionless parameter of sandwiched structure the break elongation was improved and was at maximum for the maximum T_2_ thickness. Similarly, for the TAT sample as the T_2_ increases break elongation decreases significantly for flexural tested specimens. Figure 9b shows the contour plots for the flexural strength of specimens. From plot 9b, it may be observed that as the thickness T_2_ of the sample increases in ATA samples up to 1.10 mm, the flexural strength increases, and after that the further increase of thickness T_2_ in dimensionless quantity causes the flexural strength to decrease. This may be due to the fact that a further increase in the T_2_ (above the level 1.10 mm) in the ATA sample reduced the load-resisting capability of ABS due to the presence of thin layers of ABS than the TPU layer. The opposite result was observed for TAT samples; as the T_2_ increased, the sample flexural strength increased as the layer thickness of the ABS in the TAT sample increased by a significant amount. 

**Figure 10:**
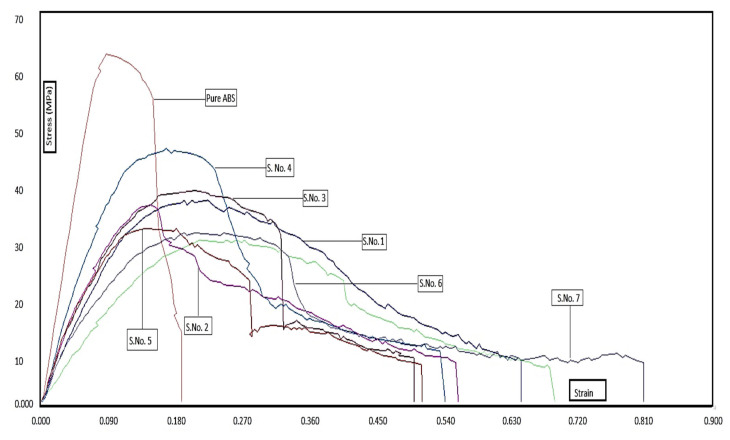
Stress vs. strain plot for ABS and ATA specimens.

**Figure 11:**
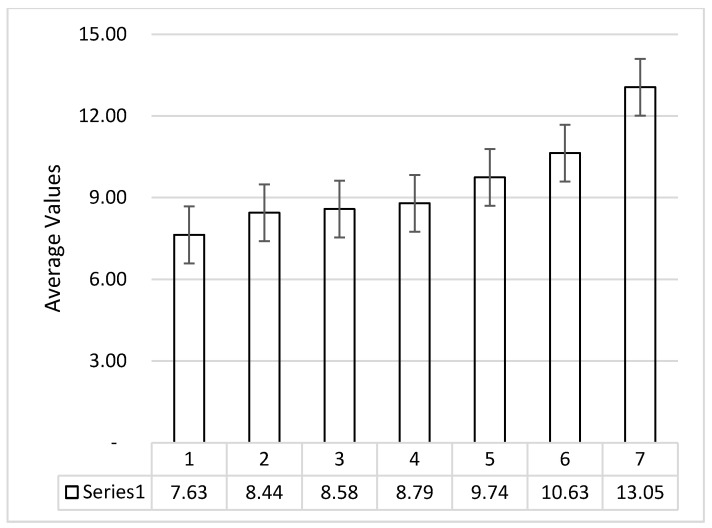
Error bar graph for flexural strength of TAT samples obtained from UTM testing.

Figure 10 shows the stress vs. strain plot for the eight samples of ABS and ATA specimens, respectively. From the stress vs. strain diagram, it may be observed that the samples with a high thickness ratio of TPU held large break elongation and strain values. However, the large thickness of the TPU layer inside the ABS structure has significantly reduced the flexural strength of specimens. Similar to the ATA specimens, the TAT specimens were also tested for three samples for each group and setting so that the error bar for the tested samples may be reported for the study. Figure 11 shows the error graph of the processed data for TAT specimens obtained from UTM testing. The average standard deviation for the data of flexural strength of TAT specimens was observed to be 1.47. Similarly, the standard deviation for the strain and Young’s modulus was observed to be valued at 0.06 and 0.28, respectively.

### 4.3. Starin Energy Absorbed per Unit Volume/Modulus of Toughness for ATA Samples

**Figure 12:**
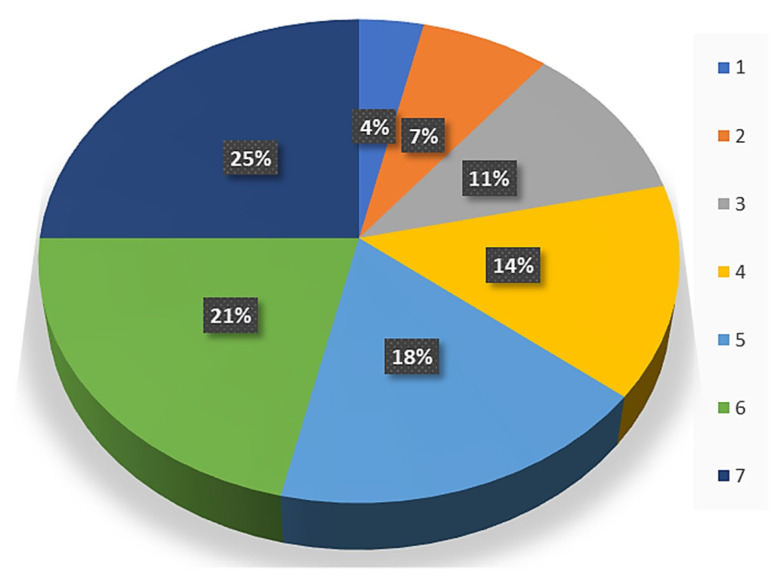
Strain absorbance capacity of the ATA specimens.

From the stress vs. strain plot, it has been ascertained that as the thickness of the inner layer of TPU increased the strain-absorbing capacity per unit volume (modulus of toughness) of the sample was increased by a significant amount. Table 7 shows the modulus of toughness for the ATA samples. From the table, it may be observed that there is a significant increase in the strain-absorbing capacity per unit volume of the samples as TPU thickness inside the ABS increased. Figure 12 shows the variation in the strain absorbance capacity per unit volume for the different ATA samples calculated based on Table 7. From Table 7, it may be observed that maximum strain absorbance per unit volume among ATA specimens exhibited by sample 7 (5.23 MPa) with the highest T2 thickness of 1.54 mm, whereas the minimum modulus of toughness was observed for least T2 thickness value. 

## 5. Morphological Properties

### 5.1. Tool Maker Microscopic Analysis

**Figure 13:**
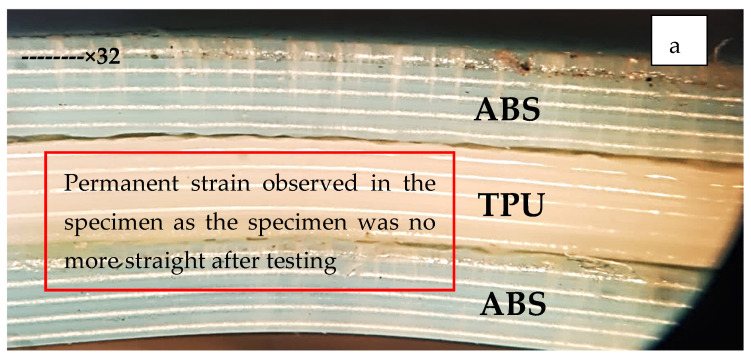

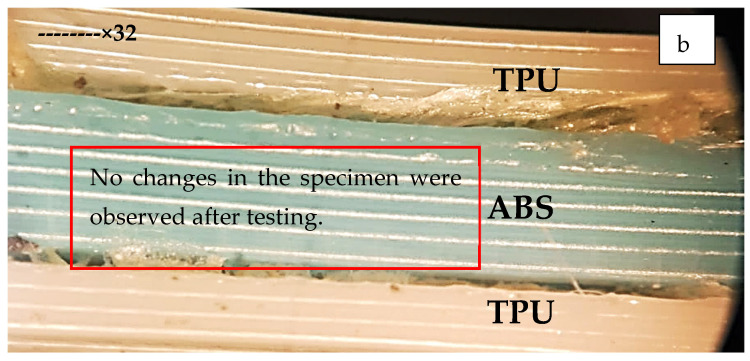
Tool Maker’s microscopic images of flexural tested specimen at ×32 for (**a**) sample 4 (ATA) and (**b**) sample 14 (TAT).

The samples tested for flexural properties were further investigated for the surface characteristics by using Tool Maker’s Microscopic tool. The lens of the Tool Maker microscope was used at an ×32 scale. From the obtained photo-micrographics of sample 4 (ATA material), a permanent change in the straightness of the sample was observed (see Figure 13a). This may be due to the fact that ABS is rigid and stiff and therefore is unable to absorb the strain energy and hence has shown permanent changes in structure. Sample 14 (TAT material) has shown no change in the specimen straightness (see Figure 13b) and has absorbed all of the strain energy applied through the UTM setup. This may be due to the fact that ABS was inside the TPU sandwiched structure and was present at the neutral axis, which is least bothered by external bending forces given by the UTM testing machine.

### 5.2. Porosity Analysis

**Figure 14:**
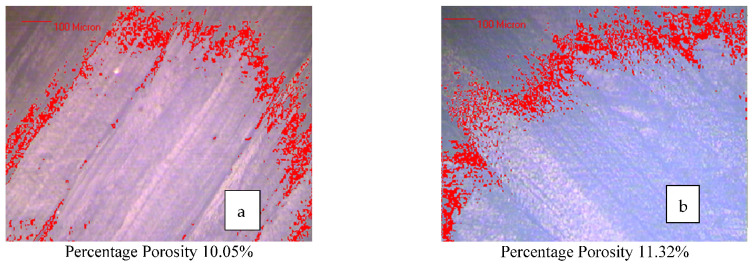
Porosity analysis for (**a**) sample 4 (ATA material specimen) and (**b**) sample 14 (TAT material specimen).

The samples were tested for porosity analysis to consider the factor of voids between the layers concerning the mechanical properties shown by the two different material specimens. For porosity analysis, a 100-micron scale in metallurgical image analysis setup (MIAS) and ASTM B276 standard was used. After selecting the area for porosity analysis for the samples, it was observed that there was no significant difference between the porosity values of the specimens, as sample 4 and sample 14 held 10% and 11.5% porosity, as shown by Figure 14a,b respectively. This may be due to the fact that each sample of TAT and ATA specimens was 3D printed on 100% infill density and was fixed in a sandwiched structure by using the epoxy and the same conditions of fixation. The porosity analysis has been performed to analyze any anomalies between the joined layers of ABS and TPU using epoxy resin. The 3D printed specimen of ABS and TPU were not under consideration for porosity analysis due to the similar conditions used during 3D printing (100% infill density). The porosity analysis has ascertained that the joined layer porosity has no significant effect on the ATA and TAT specimen-related properties.

## 6. Conclusions

The current research work highlighted the laminated object manufacturing technique for 3D printed flexural specimens of ABS and TPU. The specimens were grouped into two categories, ATA and TAT, depending on the material sandwiched inside the other material. The mechanical and morphological properties of the prepared samples were investigated, and the following major conclusions are drawn from the study.

1. ATA-based samples held greater flexural strength than TAT laminated manufactured samples. This may be due to the presence of ABS material in the outer layers of the specimen, which plays a role in compression and expansion under flexural loading, whereas the TPU was present largely at the neutral axis of the specimen for ATA-based specimens.
2. Reduction in flexural strength was significant along with the increment in the peak elongation and break elongation of the samples. For sample 4 (best among ATA samples), the percentage increase in the break elongation was observed to be 187%. In sample 7 (S.No. 7, Table 5), there was a 327% significant increment in the break elongation in comparison to pure ABS specimen but it demonstrated poor flexural strength.
3. Similarly, for the TAT-based specimen, flexural strength improved significantly from approximately 6.8 MPa to 13 MPa, which has shown a nearly 92% increase in the flexural strength in comparison to pure TPU specimen.
4. The morphological testing Tool Maker’s microscopic analysis has supported the observed trends of mechanical behavior of ATA and TAT samples, whereas from porosity analysis, it may be concluded that the porosity between the laminated layers has no significant role to play in flexural properties.

**Figure 15:**
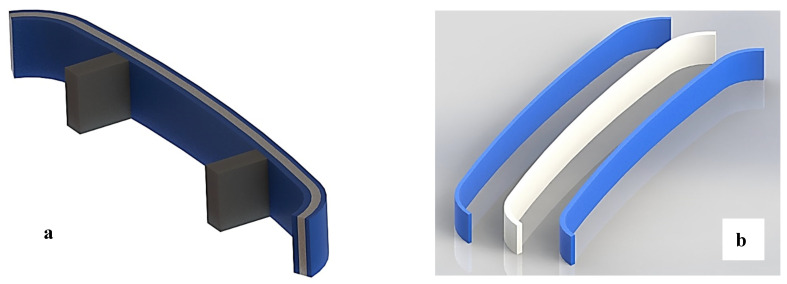
An application where tested samples of ATA may be used. (**a**) Car front bumper assembled view, and (**b**) exploded view (views taken from Solidworks software tool).

***Potential applications of the tested functionally graded specimen*.** The prepared, functionally graded material has shown a large-scale improvement in peak and break elongation of the samples; thus, the tested specimen of ATA may be used in applications for which the sample needs a large peak and break elongation along with high flexural strength, such as crash applications in automotive components, especially on car bumpers. The TAT-based specimens may be used on stairs to reduce the chances of foot slippage and can avoid accidents. Figure 15 shows the potential application of the sandwiched structure of ATA specimens designed by using the Solidworks software tool.

## Figures and Tables

**Table 1:** Previous studies performed based on LOM.

S.No.	Previous Work	Work Performed	Current Work Focused Area
1	Pilipovic et al. [36]	LOM manufacturing of paper, polymer, and metal sheet by varying the joining or working axis such as P_xy_, P_yz_, P_zx_ PVC material single polymeric matrix	(a) Sandwiched structure (b) Epoxy resins used to join the polymer (c) ABS and TPU polymeric matrix
2	Dermeik et al. [37]	A detailed evaluation of the applicability of LOM in the near net shaping ceramic-based materials. Adjustments for the LOM process and extensions of the LOM machine configuration can improve the economic feasibility its operation	Sandwiched structure of polymeric material of ABS and TPU to evaluate the effect of stiffness and flexible matrix of polymeric materials combined into one functional prototype
3.	Klosterman et al. [38]	Examines interfacial issues that arise when fabricating ceramic (SiC/SiC) and polymer matrix (glass/epoxy) composites using a novel, fully automated rapid prototyping method called laminated object manufacturing (LOM)	Deals with mechanical and morphological properties of multimaterial component of ABS and TPU; interfacial characteristics are not investigated for the present study
4	Wu et al. [39]	Three-layer sandwiched structure of polystyrene (PS) and poly(vinyl-carbazole) (PVK) as matrix layer for graphene sheets Shape memory effect study	Sandwiched structure of polymeric material of ABS and TPU No foreign infillers to alter the basic properties Deals with laminate characterization for mechanical and morphological properties only

**Table 2:** Fixed 3D printing parameters used for sample preparation.

S.No.	Printing Parameters	Values
1	Bed temperature	(a) For TPU: 50 °C (b) For ABS: 80 °C
2	Part Cooling Fan Intensity	For ABS: 0% For TPU: 50%
3	Nozzle temperature	(a) For TPU: 230 °C (b) For ABS: 240 °C
4	Infill percentage	100%
5	Surface fill	Line at 45°
6	Printing speed	40 mm/s
7	Number of perimeters	3
8	Layer height	0.12 mm
9	Layer width	0.4 mm

**Table 3:** Design of experimentation for T/T1 ratio criteria.

S.No.	T/T1	T1	T2	Material Combination
1	2.25	1.47	0.36	ATA (ABS: TPU: ABS)
2	2.5	1.32	0.66	
3	2.75	1.20	0.90	
4	3	1.10	1.10	
5	3.25	1.02	1.27	
6	3.5	0.94	1.41	
7	3.75	0.88	1.54	
8	2.25	1.47	0.36	TAT (TPU: ABS: TPU)
9	2.5	1.32	0.66	
10	2.75	1.20	0.90	
11	3	1.10	1.10	
12	3.25	1.02	1.27	
13	3.5	0.94	1.41	
14	3.75	0.88	1.54	

Samples printed for T1: 2; samples printed for T2: 1 (to make sandwiched structure); ATA: ABS: TPU: ABS and; TAT: TPU: ABS: TPU; T: total sample thickness (total thickness of the standard sample = 2 × T1 + T2).

**Table 4:** Flexural properties of pure ABS and TPU specimen.

Sample	PL (N)	PE (mm)	BL (N)	BE (mm)	FS (MPa)	FBS (MPa)
ABS	73	5.46	65.7	11.34	64.16	57.74
TPU	12.57	55.02	11.31	55.02	6.8	5.7

**Table 5:** Flexural testing results for ATA (ABS: TPU: ABS) specimens.

S. No.	Sample	PL (N)	PE (mm)	BL (N)	BE (mm)	FS (MPa)	FBS (MPa)
1	ATA	42.1	11.34	37.89	38.64	37	33.3
2	ATA	39.3	15.12	35.87	35.37	36.5	34.5
3	ATA	44.6	12.6	40.14	30.03	39.2	35.28
4	ATA	52.9	10.08	47.61	32.55	46.49	41.84
5	ATA	41.1	8.4	36.99	33.6	36.12	32.51
6	ATA	36.7	9.45	33.03	30.66	32.26	29.03
7	ATA	35.7	13.44	32.13	48.51	31.38	28.24

PL: Peak Load; PE: Peak Elongation; BL: Break Load; BE: Break Elongation; FS: Flexural Strength; FBS: Flexural Break Strength. Note: All values are average values for 3 samples of each setting.

**Table 6:** Flexural testing results for TAT (TPU: ABS: TPU) specimens (sample no 8–14).

S. No.	Sample	PL (N)	PE (mm)	BL (N)	BE (mm)	FS (MPa)	FBS (MPa)
8	TAT	8.9	40.5	7.8	53.13	7.5	6.9
9	TAT	9.9	36.91	4.41	48.85	8.31	7.1
10	TAT	5.8	35.23	5.22	46.69	8.45	7.59
11	TAT	7.8	34.6	7.02	44.26	8.66	8.01
12	TAT	9.8	33.65	8.82	41.79	9.61	8.75
13	TAT	9.3	32.76	8.37	40.15	10.50	9.36
14	TAT	14.7	30.6	13.23	39.05	12.92	11.63

**Table 7:** Strain energy is absorbed by ATA specimens (area under the curve for stress vs. strain).

S.No.	Flexural Strength (MPa)	Strain	Modulus of Toughness/Strain Energy Absorbance per Unit Volume (MPa)
1	7.5	0.65	2.437
2	8.31	0.57	2.368
3	8.45	0.49	2.070
4	8.66	0.54	2.338
5	9.61	0.47	2.258
6	10.5	0.64	3.360
7	12.92	0.81	5.232

## Data Availability

Not applicable.
